# Associations of Dietary Copper, Selenium, and Manganese Intake With Depression: A Meta-Analysis of Observational Studies

**DOI:** 10.3389/fnut.2022.854774

**Published:** 2022-03-15

**Authors:** Jun Ding, Yi Zhang

**Affiliations:** ^1^Changsha Social Work College, Changsha, China; ^2^Department of Orthopaedics, Xiangya Hospital, Central South University, Changsha, China; ^3^National Clinical Research Center for Geriatric Disorders, Xiangya Hospital, Central South University, Changsha, China

**Keywords:** dietary copper intake, dietary selenium intake, dietary manganese intake, depression, meta-analysis, observational studies

## Abstract

**Objective:**

To comprehensively summarize the evidence on the associations of dietary copper, selenium, and manganese intake with depression based on a meta-analysis of observational studies.

**Methods:**

The electronic database of PubMed, Web of Science, and Embase were searched up to January 7, 2022, for observational studies on the associations of dietary copper, selenium and manganese intake with depression (no restriction was set for the initiate time). The pooled relative risk (RR) of depression for the highest vs. lowest dietary copper, selenium, and manganese intake category were calculated.

**Results:**

A total of 11 observational studies (61,430 participants) were identified as meeting the inclusion criteria. Specifically, five studies were related to the dietary copper intake. The overall multi-variable adjusted RR demonstrated that dietary copper intake was inversely associated with depression (RR = 0.63, 95% CI: 0.52–0.76; *P* < 0.001; *I*^2^ = 2.4%). With regard to the dietary selenium intake, six studies were identified for meta-analysis. The overall multi-variable adjusted RR showed that dietary selenium intake was also negatively associated with depression (RR = 0.63, 95% CI: 0.54–0.74; *P* < 0.001; *I*^2^ = 37.8%). In addition, four studies were specified for the dietary manganese intake, and the overall multi-variable adjusted RR indicated a negative relationship between dietary manganese intake and depression (RR = 0.71, 95% CI: 0.58–0.86; *P* < 0.001; *I*^2^ = 0.0%).

**Conclusions:**

Our results suggest a negative relationship between dietary copper, selenium and manganese intake and depression, respectively. However, due to the limited prospective evidence, our results are restricted to cross-sectional design that precludes causal relationships. More well-designed prospective cohort studies are still needed.

## Introduction

Depression is one of the most common global mental disorders (affecting females twice as much as males) (1), which usually presents as exhaustion, sadness, and lack of interest in daily activities (2). As a global burden of disease affecting ~300 million people (3), depression is estimated to be the leading cause of disability worldwide by 2030 (4). Nevertheless, the current treatment for depression may be limited to the following issues: costly pharmacotherapy, adverse side effects and unsatisfactory curative effect (5). Emerging evidence suggests that dietary factors are associated with depression (6, 7). Thus, the identification of modifiable dietary factors for depression appears to be an important step in its clinical prevention and management.

Micronutrients are important factors for cellular biochemical functions. Among them, copper, selenium, and manganese are considered to be significant ones. As a component of extracellular superoxide dismutase (8), copper is essential for iron uptake and signaling in energy metabolism, reactive oxygen species detoxification and eukaryotic organisms (9). In addition, copper plays a significant role in signaling involving mitophagy, bioenergetics, and dynamics and mitochondrial function, which determine cellular fate by metabolic reprogramming (9). In addition, selenium is severed as an essential micronutrient that maintain the different cellular functions, such as immune-endocrine function and signaling transduction pathways (10). Moreover, selenium incorporates into selenoproteins and selenium-dependent enzymes (e.g., glutathione peroxidases), which is closely related to intracellular redox regulation and modulation (11). On the other hand, as another essential nutrient for the body, manganese is an important component of manganese superoxide dismutase (MnSOD, SOD-2), which is the primary antioxidant enzyme that protects cells from oxidative stress (catalyze the dismutation of superoxide to hydrogen peroxide and oxygen in the mitochondria) (12). Since the oxidative stress is considered to play a significant role in the pathophysiology of depression (13, 14), the dietary copper, selenium, and manganese intake is considered to be beneficial to depression.

As far as we know, a number of observational studies have been employed to investigate the associations of dietary copper, selenium, and manganese intake with depression (15–25). However, their results are still conflicting. Thus, this meta-analysis of observational studies is employed to investigate the issues further. It is hypothesized that the dietary copper, selenium, and manganese intake is inversely associated with depression, respectively.

## Materials and Methods

### Search Strategy

Our meta-analysis was performed according to the Preferred Reporting Items for Systematic Reviews and Meta-analyses (PRISMA) guidelines (26). The electronic database of PubMed, Web of Science and Embase were searched up to January 7, 2022 (no restriction was set for the initiate time) by using a combination of keywords that related to depression (“depression,” “depressive”), copper (“copper”), selenium (“selenium”), and manganese (“manganese”). No language restriction was set in the search strategy. We screened the titles and abstracts of all articles, and then read the full articles to identify the eligible studies.

### Study Selection

Two researchers reviewed the titles, abstracts, and full texts of all retrieved studies independently. Disagreements were resolved by discussions. The inclusion criteria were listed as follows: (1) observational studies; (2) the associations of dietary copper, selenium and manganese intake with depression; and (3) relative risk (RR) or odds ratio (OR) with 95% confidence interval (CI) was reported. The exclusion criteria were listed as follows: (1) duplicated or irrelevant articles; (2) reviews, letters, or case reports; (3) randomized controlled trials; and (4) non-human studies.

### Data Extraction

The data was extracted by two researchers independently, and disagreements were resolved by discussions. The information about the first author and year of publication, location, age, sex, sample size, study design, adjustments, exposure assessment, category of exposure, effect estimates, and diagnostic criteria of depression, was collected. The corresponding effect estimates of depression with 95% CIs for the highest vs. lowest dietary copper, selenium and manganese intake category were extracted (adjusted for the maximum number of confounding variables).

### Quality Assessment

The Newcastle-Ottawa (NOS) criteria for non-randomized studies was employed to assess the quality of each included study. NOS is based on three broad perspectives: (1) the selection process of study cohorts; (2) the comparability among different cohorts; (3) the identification of exposure or outcome of study cohorts. Disagreements with respect to the methodological quality were resolved by discussion.

### Statistical Analyses

The RR for depression were the outcome measures in this meta-analysis. The *I*^2^ statistic, which measures the percentage of total variation across studies due to heterogeneity, was examined (*I*^2^ > 50% was considered heterogeneity). If significant heterogeneity was observed among the studies, the random-effects model was used; otherwise, the fixed effects model was accepted. Begg's test was employed to assess the publication bias (27). Moreover, subgroup analysis for sex, geographical region, sample size, diagnostic criteria of depression, exposure assessment, population, and study design were employed.

## Results

### Study Identification and Selection

**Figure 1:**
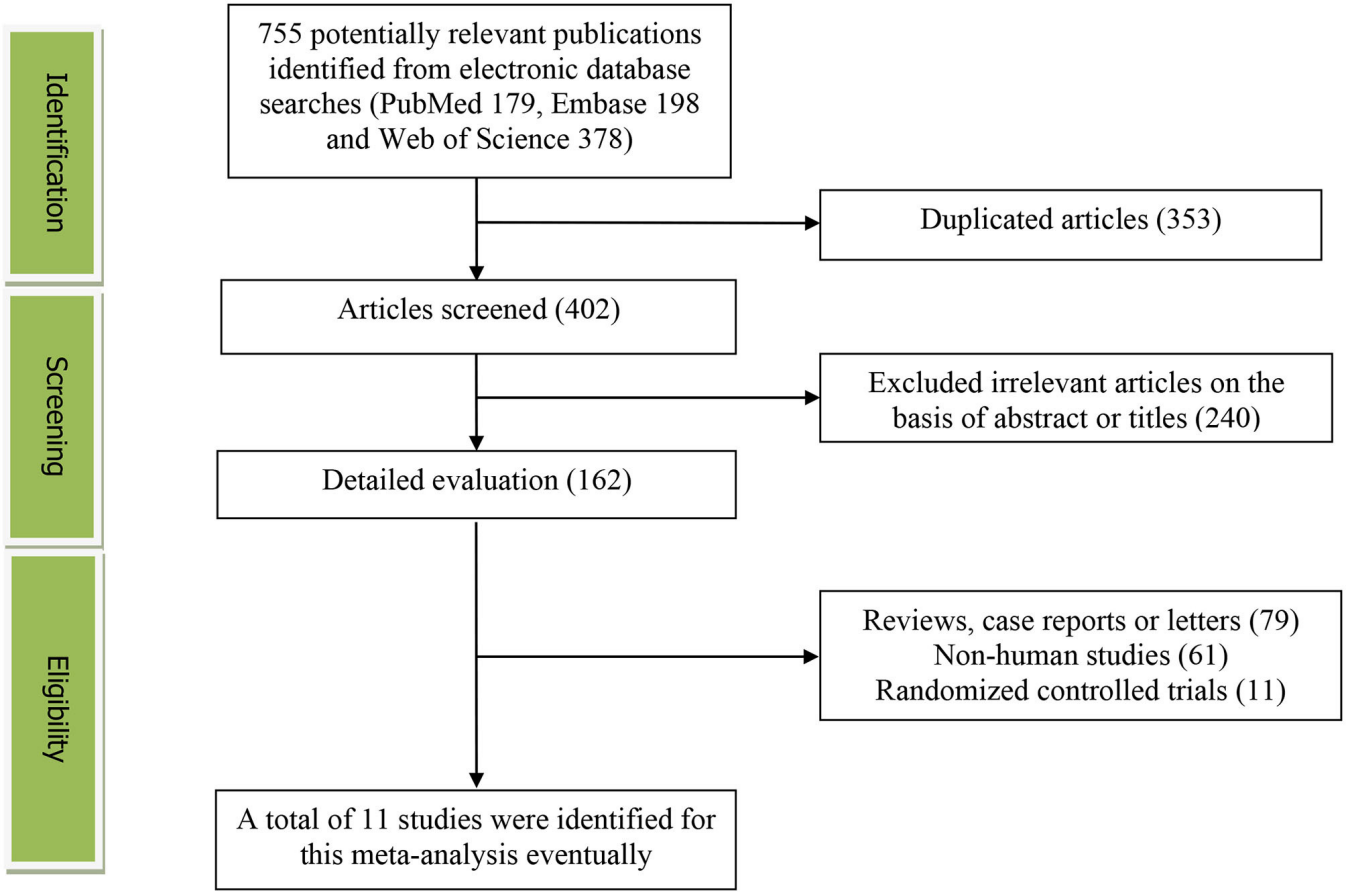
The detailed flow diagram of the study identification and selection in this meta-analysis.

The detailed flow diagram of the study identification and selection were presented in Figure 1. Initially, a total of 755 potentially relevant articles (179 for PubMed, 198 for Embase, and 378 for Web of Science) were retrieved during the literature search. After eliminating 353 duplicated articles, 402 articles were screened according to the titles and abstracts, and then, 240 irrelevant studies were excluded. Thereafter, 79 reviews, case reports or letters, 61 non-human studies, 11 randomized controlled trials studies were removed. Eventually, 11 studies were selected for this meta-analysis (15–25).

### Study Characteristics

The main characteristics of the included studies were presented in Table 1. These studies were published between 2012 and 2022. Among them, five studies were performed in Asian countries [Japan (17, 21, 22), China (23) and Korea (16)], and the other six ones were from US (19, 20), Brazil (24, 25), Spain (18), and Australia (15). Four articles included only females (15–17, 23), and the other seven studies recruited both males and females (18–22, 24, 25). With regard to the study design, nine studies were cross-sectional/case-control (16, 17, 19–25) and two ones were prospective cohort (15, 18) studies. The sample size ranged from 316 to 14,834 for a total number of 61,430. The dietary micronutrients were assessed by food-frequency questionnaire (FFQ) in eight studies (15–18, 21–23, 25), and recall method in three studies (19, 20, 24). The diagnostic criteria of depression were Diagnostic and Statistical Manual of Mental Disorders-IV (DSM-IV) (15, 18), Patient Health Questionnaire-9 (PHQ-9) (19, 20), Center for Epidemiological Studies Depression Scale (CES-D) (17, 23), Beck Depression Inventory (BDI) (16), Kessler's six-item psychological distress scale (K6) (21), Geriatric Depression Scale (GDS) (22), Mini-International Neuropsychiatric Interview (MINI) (24), and Clinical Interview Schedule Revised (CIS-R) (25), respectively.

**Table 1:** Characteristics of the individual studies included in this meta-analysis.

**References**	**Location**	**Age (years)**	**Sex**	**Sample size**	**Study design**	**Adjustments**	**Exposure assessment**	**Category of exposure**	**Effect estimates**	**Diagnostic criteria of depression**	**NOS**
Pasco et al. (15)	Australia	20–89	Female	316	Cohort	Age and socioeconomic status	FFQ	Selenium Low intake High intake	1.00 0.34 (0.12, 0.96)	DSM-IV	7
Kim et al. (16)	Korea	12–18	Female	849	Case-control	Menstrual regularity and energy	FFQ	Copper Tertile 1 Tertile 2 Tertile 3	1.00 0.78 (0.48, 1.38) 0.41 (0.17, 0.96)	BDI	7
Miyake et al. (17)	Japan	31	Female	1,745	Cross-sectional	Age, gestation, region of residence, number of children, family structure, history of depression, family history of depression, smoking, secondhand smoke exposure at home and at work, employment, household income, education, BMI, intake of saturated fatty acids, eicosapentaenoic acid plus docosahexaenoic acid, calcium, vitamin D and isoflavones	FFQ	Copper Quartile 1 Quartile 2 Quartile 3 Quartile 4 Manganese Quartile 1 Quartile 2 Quartile 3 Quartile 4	1.00 0.74 (0.57, 0.96) 0.80 (0.60, 1.06) 0.73 (0.51, 1.05) 1.00 0.93 (0.72, 1.19) 0.94 (0.73, 1.21) 0.74 (0.56, 0.97)	CES-D	6
Sánchez-Villegas et al. (18)	Spain	38	Both	13,983	Cohort	Sex, age, physical activity, BMI, energy intake, special diets, smoking, alcohol intake and prevalence of CVD, HTA, or T2DM	FFQ	Selenium Inadequacy Adequacy	1.00 0.78 (0.57, 1.07)	DSM-IV	8
Li et al. (19)	US	>18	Both	14,834	Cross-sectional	Age, gender, BMI, race, educational level, smoking status, family income, work activity, recreational activity, hypertension, diabetes, and total daily energy intake	Recall method	Copper Quartile 1 Quartile 2 Quartile 3 Quartile 4 Selenium Quartile 1 Quartile 2 Quartile 3 Quartile 4	1.00 0.81 (0.65, 1.03) 0.78 (0.62, 0.98) 0.68 (0.49, 0.94) 1.00 0.69 (0.53, 0.91) 0.52 (0.39, 0.69) 0.46 (0.32, 0.67)	PHQ-9	8
Ghimire et al. (20)	US	>18	Both	7,725	Cross-sectional	Age, sex, race ethnicity, marital status, educational status, family poverty income ratio, BMI, smoking, alcohol use, physical activity, and use of dietary supplements, diabetes, kidney disease, cancer, and heart disease and total energy intake	Recall method	Selenium Quintile 1 Quintile 2 Quintile 3 Quintile 4 Quintile 5	1.00 0.64 (0.48, 0.85) 0.69 (0.49, 0.96) 0.57 (0.36, 0.90) 0.60 (0.39, 0.94)	PHQ-9	8
Nakamura et al. (21)	Japan	18–79	Both	2,089	Cross-sectional	Age, sex, smoking, alcohol drinking, BMI, shift work, and intake of Vitamin C, B6, B12, folic acid, PUFA, medications for hypertension, hyperlipidemia, and diabetes	FFQ	Copper Quartile 1 Quartile 2 Quartile 3 Quartile 4 Manganese Quartile 1 Quartile 2 Quartile 3 Quartile 4	1.00 0.60 (0.31, 1.16) 0.52, (0.28, 0.97) 0.43 (0.22, 0.84) 1.00 0.56 (0.27, 1.16) 0.51 (0.27, 0.96) 0.51, (0.24, 1.08)	K6	7
Nguyen et al. (22)	Japan	>65	Both	1,423	Cross-sectional	Age, BMI, living status, having a job status, married status, smoking status, alcohol consumption, total energy, hypertension, diabetes, and hyperlipidemia	FFQ	Male Copper Quartile 1 Quartile 2 Quartile 3 Quartile 4 Manganese Quartile 1 Quartile 2 Quartile 3 Quartile 4 Female Copper Quartile 1 Quartile 2 Quartile 3 Quartile 4 Manganese Quartile 1 Quartile 2 Quartile 3 Quartile 4	1.00 0.78 (0.42, 1.42) 0.78 (0.43, 1.41) 0.78 (0.42, 1.42) 1.00 1.21 (0.67, 2.18) 1.51 (0.84, 2.71) 0.83 (0.45, 1.53) 1.00 0.81 (0.49, 1.34) 0.61 (0.36, 1.02) 0.43 (0.25, 0.77) 1.00 1.08 (0.65, 1.82) 0.80 (0.47, 1.37) 0.75 (0.43, 1.30)	GDS	7
Li et al. (23)	China	42–52	Female	2,993	Cross-sectional	Energy intake, saturated fatty acids intake, unsaturated fatty acids intake, *n*−3 PUFA intake, vitamin B6 intake, vitamin B12 intake, vitamin C intake, vitamin D intake, calcium intake, copper intake, zinc intake, age, race/ethnicity, education, income, financial strain, physical activity, BMI, VMS, chronic stress, use of antidepressant, estradiol, testosterone, and SHBG	FFQ	Manganese Early perimenopausal Quartile 1 Quartile 2 Quartile 3 Quartile 4 Premenopausal Quartile 1 Quartile 2 Quartile 3 Quartile 4	1.00 0.87 (0.58, 1.31) 0.79 (0.49, 1.27) 0.80 (0.46, 1.39) 1.00 0.97 (0.66, 1.43) 0.71 (0.45, 1.11) 0.51 (0.29–0.91)	CES-D	7
Almeida et al. (24)	Brazil	18–59	Both	736	Cross-sectional	Gender, marital status, socioeconomic class, alcohol consumption, and pesticide poisoning	Recall method	Selenium Low intake High intake	1.00 0.46 (0.24, 0.90)	MINI	7
Ferriani et al. (25)	Brazil	35–74	Both	14,737	Cross-sectional	Age, race, total cholesterol, HDL cholesterol, systolic blood pressure, antihypertensive drug, diabetes, and smoking, cardiovascular disease, physical activity, and calorie	FFQ	Selenium Quintile 1 Quintile 2 Quintile 3 Quintile 4 Quintile 5	1.00 0.88 (0.69, 1.12) 0.80 (0.62, 1.03) 0.76 (0.59, 0.98) 0.72 (0.56, 0.94)	CIS-R	8

*BMI, Body mass index; FFQ, Food frequency questionnaire; CVD, Cardiovascular disease; HDL, High density lipoprotein; HTA, Hypertension; T2DM, Type 2 diabetes mellitus; PUFA, Polyunsaturated fatty acid; VMS, Vasomotor symptoms; SHBG, Sex hormone binding globulin; DSM-IV, Diagnostic and Statistical Manual of Mental Disorders-IV; PHQ-9, Patient Health Questionnaire-9; CES-D, Center for Epidemiological Studies Depression Scale; BDI, Beck Depression Inventory; K6, Kessler's six-item psychological distress scale; GDS, Geriatric Depression Scale; MINI, Mini-International Neuropsychiatric Interview; CIS-R, Clinical Interview Schedule Revised*.

### RR of Depression for the Highest vs. Lowest Dietary Copper Intake Category

**Figure 2:**
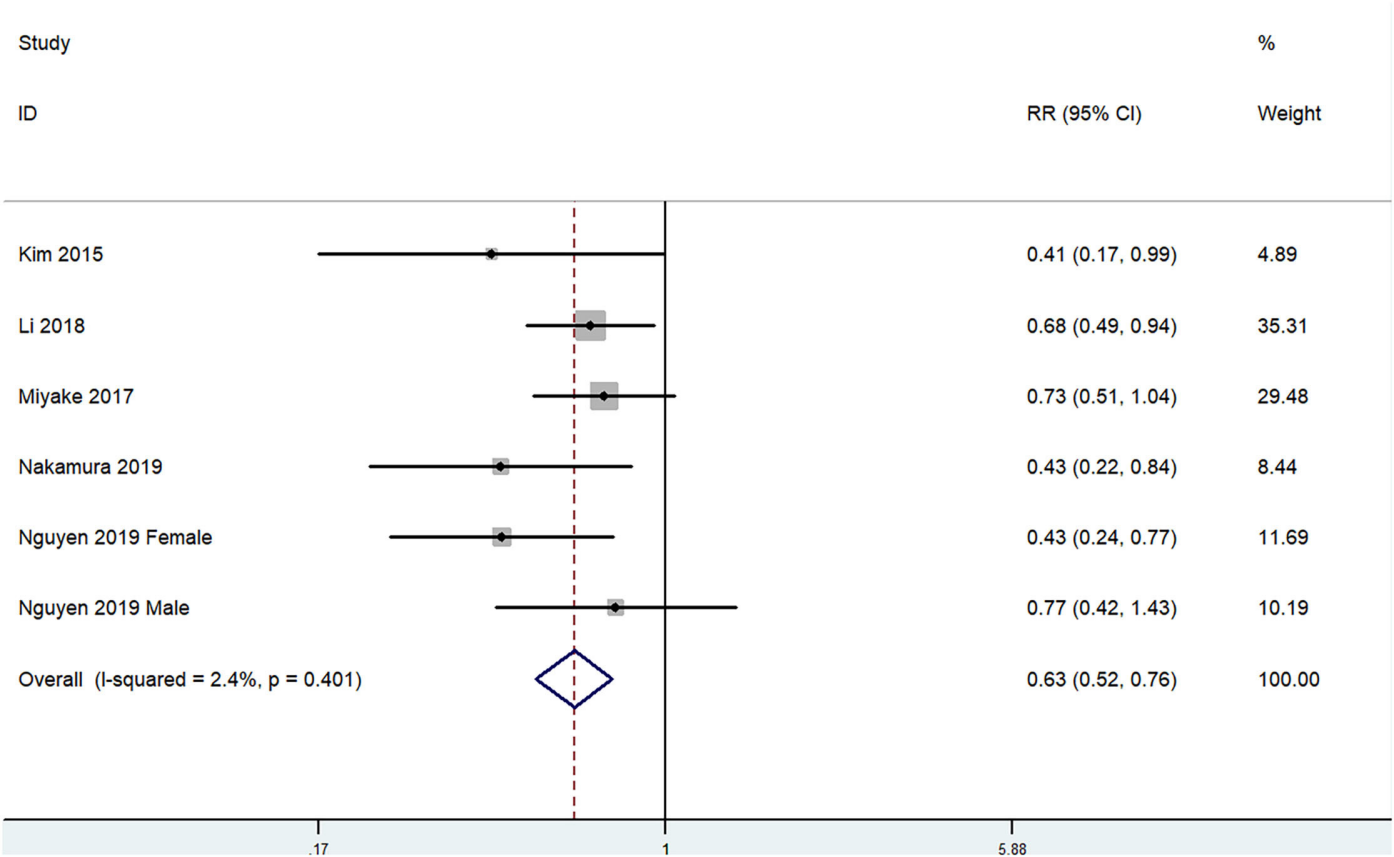
Forest plot of meta-analysis: overall multi-variable adjusted RR of depression for the highest vs. lowest category of dietary copper intake.

The overall multi-variable adjusted RR showed that the dietary copper intake was inversely associated with depression (RR = 0.63, 95% CI: 0.52–0.76; *P* < 0.001; Figure 2). No substantial level of heterogeneity was obtained among various studies (*P* = 0.401, *I*^2^ = 2.4%). No evidence of publication bias existed according to the Begg's rank-correlation test (*P* = 0.707). Table 2 presented the results of subgroup analysis. The above findings were confirmed in female (RR = 0.60, 95% CI: 0.40–0.80; *P* < 0.001), but not in male (RR = 0.64, 95% CI: 0.36–1.11).

**Table 2:** Subgroup analysis of depression for the highest vs. lowest dietary copper intake category.

**Stratification**	**Number of studies**	**Pooled RR**	**95% CI**	***P*** **-value**	**Heterogeneity**
All studies	5	0.63	0.52, 0.76	*P* <0.001	*P* = 0.40; *I*^2^ = 2%
**Sex**					
Male	1	0.77	0.42, 1.43	/	/
Female	3	0.60	0.40, 0.80	*P* <0.001	*P* = 0.21; *I*^2^ = 36%
**Geographical region**					
Asia	4	0.60	0.47, 0.76	*P* <0.001	*P* = 0.31; *I*^2^ = 16%
Non-Asia	1	0.68	0.49, 0.94	/	/
**Sample size**					
<2,000	3	0.63	0.49, 0.82	*P* <0.001	*P* = 0.30; *I*^2^ = 18%
>2,000	2	0.62	0.46, 0.84	*P* = 0.002	*P* = 0.23; *I*^2^ = 31%
**Exposure assessment**					
FFQ	4	0.60	0.47, 0.76	P <0.001	*P* = 0.31; *I*^2^ = 16%
Recall method	1	0.68	0.49, 0.94	/	/
**Population**					
Adolescent	1	0.41	0.17, 0.99	/	/
Middle aged and elderly	4	0.64	0.52, 0.78	*P* <0.001	*P* = 0.38; *I*^2^ = 4%

*RR, Relative risk; CI, Confidence interval; FFQ, Food frequency questionnaire*.

### RR of Depression for the Highest vs. Lowest Dietary Selenium Intake Category

**Figure 3:**
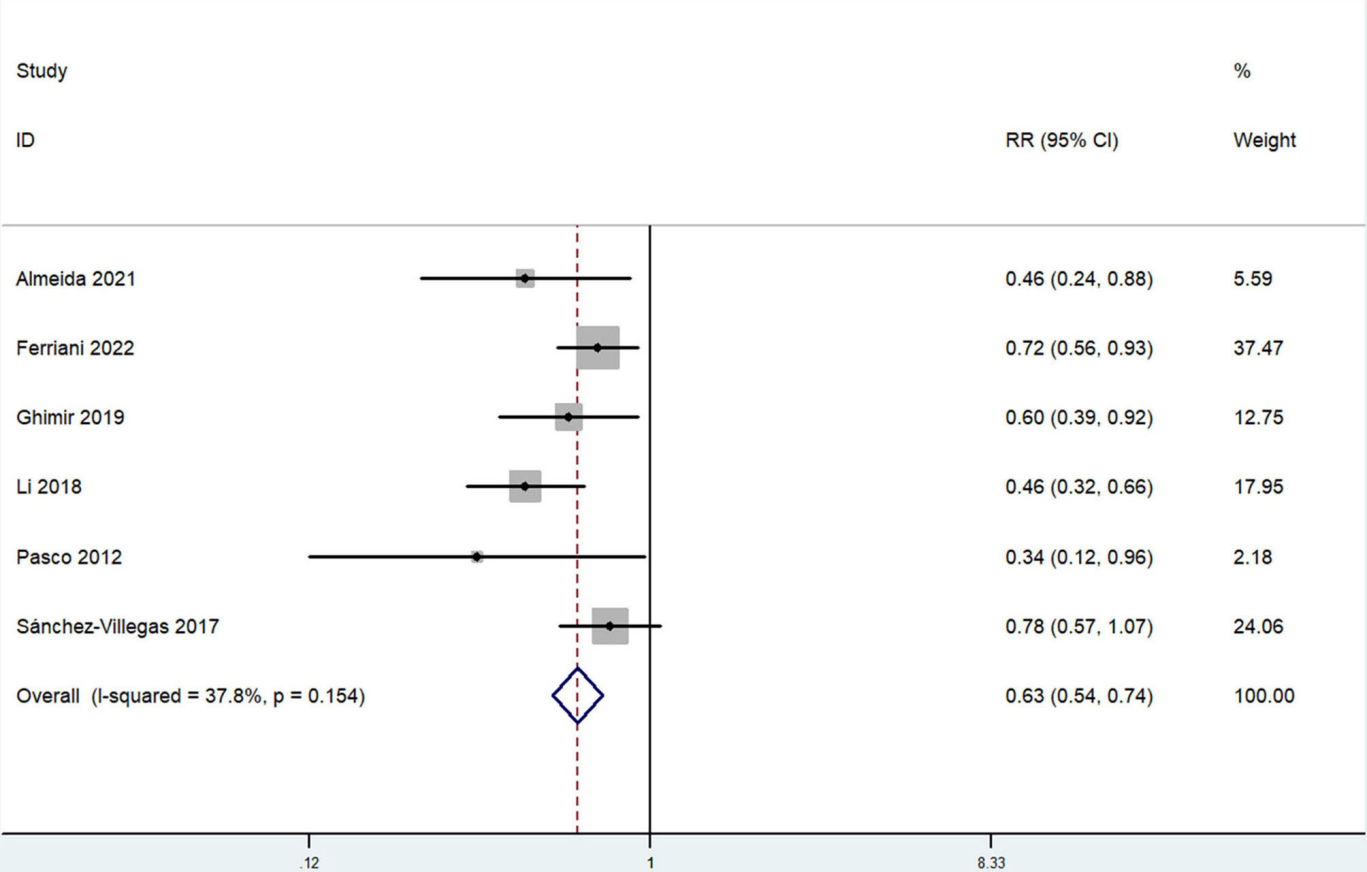
Forest plot of meta-analysis: overall multi-variable adjusted RR of depression for the highest vs. lowest category of dietary selenium intake.

The overall multi-variable adjusted RR showed that the dietary selenium intake was inversely associated with depression (RR = 0.63, 95% CI: 0.54–0.74; *P* < 0.001) (Figure 3). No substantial level of heterogeneity was obtained among various studies (*P* = 0.154, *I*^2^ = 37.8%). No evidence of publication bias existed according to the Begg's rank-correlation test (*P* = 0.260). Table 3 presented the results of subgroup analysis. The above findings were confirmed in female (RR = 0.63, 95% CI: 0.47–0.85; *P* = 0.003), PHQ-9 (RR = 0.51, 95% CI: 0.39–0.68; *P* < 0.001), cross-sectional (RR = 0.60, 95% CI: 0.51–0.72; *P* < 0.001) studies, but not in male (RR = 0.77, 95% CI: 0.42–1.43; *P* = 0.001), DSM-IV (RR = 0.60, 95% CI: 0.28–1.28; *P* = 0.19) and prospective cohort studies (RR = 0.60, 95% CI: 0.28–1.28; *P* = 0.19).

**Table 3:** Subgroup analysis of depression for the highest vs. lowest dietary selenium intake category.

**Stratification**	**Number of studies**	**Pooled RR**	**95% CI**	***P*** **-value**	**Heterogeneity**
All studies	6	0.63	0.54, 0.74	*P* <0.001	*P* = 0.15; *I*^2^ = 38%
**Sex**					
Male	1	0.64	0.36, 1.11	/	/
Female	2	0.63	0.47, 0.85	*P* = 0.003	*P* = 0.22; *I*^2^ = 33%
**Diagnostic criteria of depression**					
DSM-IV	2	0.60	0.28, 1.28	*P* = 0.19	*P* = 0.13; *I*^2^ = 55%
PHQ-9	2	0.51	0.39, 0.68	*P* <0.001	*P* = 0.36; *I*^2^ = 0%
**Geographical region**					
US	2	0.51	0.39, 0.68	*P* <0.001	*P* = 0.36; *I*^2^ = 0%
Brazil	2	0.68	0.54, 0.86	*P* = 0.001	*P* = 0.21; *I*^2^ = 37%
**Sample size**					
<2,000	2	0.42	0.24, 0.73	*P* = 0.002	*P* = 0.63; *I*^2^ = 0%
>2,000	4	0.66	0.56, 0.77	*P* <0.001	*P* = 0.14; *I*^2^ = 46%
**Exposure assessment**					
FFQ	3	0.72	0.60, 0.88	*P* = 0.001	*P* = 0.33; *I*^2^ = 11%
Recall method	3	0.51	0.39, 0.65	*P* <0.001	*P* = 0.62; *I*^2^ = 0%
**Study design**					
Cross-sectional	4	0.60	0.51, 0.72	*P* <0.001	*P* = 0.19; *I*^2^ = 36%
Cohort	2	0.60	0.28, 1.28	*P* = 0.19	*P* = 0.13; *I*^2^ = 55%

*RR, Relative risk; CI, Confidence interval; FFQ, Food frequency questionnaire; DSM-IV, Diagnostic and Statistical Manual of Mental Disorders-IV; PHQ-9, Patient Health Questionnaire-9*.

### RR of Depression for the Highest vs. Lowest Dietary Manganese Intake Category

**Figure 4:**
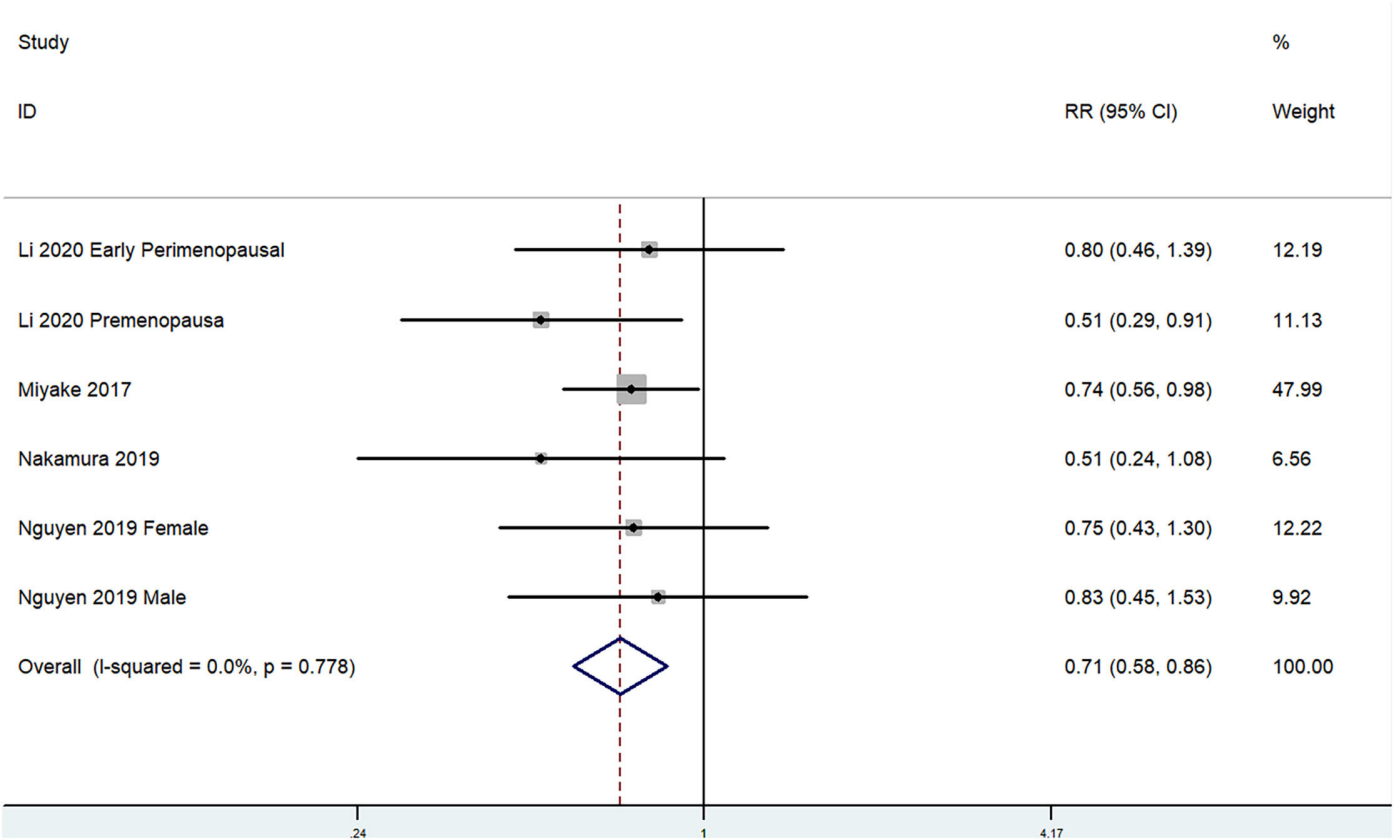
Forest plot of meta-analysis: overall multi-variable adjusted RR of depression for the highest vs. lowest category of dietary manganese intake.

The overall multi-variable adjusted RR showed that the dietary manganese intake was inversely associated with depression (RR = 0.71, 95% CI: 0.58–0.86; *P* < 0.001; Figure 4). No substantial level of heterogeneity was obtained among various studies (*P* = 0.778, *I*^2^ = 0.0%). No evidence of publication bias existed according to the Begg's rank-correlation test (*P* = 1.000). Table 4 presented the results of subgroup analysis. The above findings were confirmed in female (RR = 0.71, 95% CI: 0.58–0.88; *P* = 0.002) and CES-D (RR = 0.71, 95% CI: 0.56–0.89; *P* = 0.003) studies, but not in male (RR = 0.83, 95% CI: 0.45–1.53) and other criteria studies (RR = 0.71, 95% CI: 0.49–1.02; *P* = 0.06).

**Table 4:** Subgroup analysis of depression for the highest vs. lowest dietary manganese intake category.

**Stratification**	**Number of studies**	**Pooled RR**	**95% CI**	***P*** **-value**	**Heterogeneity**
All studies	4	0.71	0.58, 0.86	*P* <0.001	*P* = 0.78; *I*^2^ = 0%
**Sex**					
Male	1	0.83	0.45, 1.53	/	/
Female	3	0.71	0.58, 0.88	*P* = 0.002	*P* = 0.68; *I*^2^ = 0%
**Diagnostic criteria of depression**					
CES-D	2	0.71	0.56, 0.89	*P* = 0.003	*P* = 0.48; *I*^2^ = 0%
Other	2	0.71	0.49, 1.02	*P* = 0.06	*P* = 0.60; *I*^2^ = 0%
**Geographical region**					
Japan	3	0.73	0.58, 0.91	*P* = 0.005	*P* = 0.79; *I*^2^ = 0%
China	1	0.65	0.43, 0.96	/	/
**Sample size**					
<2,000	2	0.75	0.60, 0.95	*P* = 0.02	*P* = 0.95; *I*^2^ = 0%
>2,000	2	0.61	0.43, 0.87	*P* = 0.007	*P* = 0.48; *I*^2^ = 0%

*RR, Relative risk; CI, Confidence interval; FFQ, Food frequency questionnaire; CES-D, Center for Epidemiological Studies Depression Scale*.

## Discussion

A total of 11 observational studies were identified in the present meta-analysis. The pooled results demonstrated a negative relationship between dietary copper, selenium, and manganese intake and depression, respectively.

The pathophysiology of depression is involved in oxidative stress, whereas copper, selenium, and manganese are served as important antioxidants that act against oxidative stress. Copper is a cofactor of the copper/zinc superoxide dismutase, a protein located in both the cytosol and mitochondrial inner membrane space to relieve the electron transport chain-generated reactive oxygen species (9). On the other hand, copper may drive the activity of the two neurotrophic factors Brain-derived neurotrophic factor (BDNF) and nerve growth factor (NGF) (28), which further influence the activity-dependent neural plasticity and neural network (29). Indeed, experimental evidence demonstrates that low-dose copper exacerbates depression-like behavior in ApoE4 transgenic mice (30). Differently from other metals, selenium incorporates into selenoproteins (glutathione per-oxidases and thioredoxin reductases) and protects from lipoperoxidation and oxidative cell damage (the glutathione antioxidant system is implicated in the pathophysiology of mood disorders) (31). Consistently, the selenocompound 1-methyl-3-(phenylselany1)-1H-indole attenuates depression-like behavior, oxidative stress, and neuroinflammation in streptozotocin-treated mice (32). Moreover, manganese is an important component of MnSOD, an antioxidant mitochondrial metalloenzyme that protects cells from oxidative stress (12, 33). Reduced MnSOD activity due to manganese deficiency might contribute to the development of depression. These above may significantly account for the major findings of our study.

Interestingly, our findings are only confirmed in females, but not males. It suggests that some genetic sexual differences with the diet-related pathology of depression should be considered. For example, the genetic contributions of the serotonin transporter in depression may be different (34), and the process of some serotonin systems may be more apparent in females than that in males either (35). Importantly, the inverse relationship between dietary selenium intake and depression is lost in prospective cohort study, which might be attributed to the potential reversed causality (e.g., depressive subjects may consume less dietary copper, selenium and manganese due to the reduced appetite). Moreover, the diagnostic criteria of depression vary greatly among individuals, which may also influence the reliability of subgroup analysis. Overall, very small number of studies are qualified for subgroup analysis, and the corresponding results should be considered very carefully. More well-designed prospective cohort studies with sexual specification are still needed.

It should also be noted that a very recent meta-analysis study has investigated the role of selenium in depression (36). The authors fail to demonstrate any significant differences in serum selenium levels between depressive and healthy subjects. On the contrary, they find the selenium supplementation significantly reduces depressive symptoms. The inconsistent results may be explained as follow: (1) The selenium in serum may not reflect the issues of dietary selenium intake (37). In fact, demographic variables, health status, and some other factors may also influence serum selenium levels (38), and only one study has adjusted these confounding variables (39). (2) Given that the long-term exposure to low serum selenium level may impair brain function (40), the duration of low selenium intake is ignored in most studies. Importantly, their overall OR result shows no significant relationship between dietary selenium intake and depression. However, their search was performed on June 30, 2020 (updated on April 12, 2021) and two recent published studies were not specified for analysis (24, 25). Moreover, the category of exposure was unclear in one included study either (excluded in the present meta-analysis) (41). Most importantly, the effect estimates for the highest vs. lowest and lowest vs. highest (inadequate vs. adequate) exposure category was also pooled directly. Above all, our study is an important advance and supplement to their study.

Another relevant meta-analysis study has also comprehensively evaluated the relationship between body burden of copper and depression (42). They demonstrate that the blood copper level in depressive subjects is higher than that in controls, which implies that blood copper may be served as a biomarker for depression. On this basis, our study further demonstrates that dietary copper intake is inversely associated with depression either. Interestingly, Johnson et al. further found an inverse relationship between selenium level in household groundwater and depression, and GPX1 gene is related to depression risk and significantly influences the protective impact of selenium (43), which indicates a gene-environment interaction.

Although our findings may encourage to build an awareness with the collaboration between physicians and nutritionists, our results might be influenced by environmental and medical treatment factors, the interaction of multiple dietary factors, and the reversed causality (depressed individuals may have irregular/inadequate nutrition patterns that lead to nutritional inadequacy of these micronutrients intake). Moreover, the toxicity of these micronutrients should also be recognized. For instance, excess copper intake is reported to induce oxidative stress, damage to the mitochondrial, and leads to apoptosis, DNA damage and inflammatory responses (44, 45). In addition, selenium exposure is associated with increased risk for type 2 diabetes (46). Elevated selenium exposure has also been suspected to be a risk factor for the development of several neurodegenerative and neuropsychiatric diseases (47, 48). Moreover, long-term exposure to manganese may have adverse effects on mood state, neurobehavior, and peripheral neurotransmitters (49). Therefore, a careful validation by high-quality randomized controlled trial/prospective cohort study is still needed.

Our study has several strengthens. First, this is the first meta-analysis of observational studies on the associations of dietary copper, selenium, and manganese intake with depression. In addition, the included studies are analyzed based on the adjusted results and large samples. Moreover, the limited heterogeneity level may reflect a decent reliability of our results. Finally, our findings may provide significant information to better consider the dietary effects on depression. The limitations of our study should also be acknowledged. First, only two prospective cohort studies were identified due to the limited relevant literature, which precludes causal relationships (depressive subjects may consume less dietary copper, selenium, and manganese due to the reduced appetite). Second, the classification of exposure and diagnostic criteria of depression varies greatly among individuals. Third, the adjusted factors were not uniform. Fourth, the environmental and medical treatment factors are considered in few studies, their impact cannot be clearly clarified and our topic might be over-simplified (the interaction of multiple dietary factors). Last but not the lease, the circulating level of these micronutrients is not considered due to the limited evidence, and the issue of microelement deficiency cannot be addressed. These limitations may weaken the significance of our study.

## Conclusions

Our results suggest a negative relationship between dietary copper, selenium, and manganese intake and depression, respectively. However, due to the limited prospective evidence, our results are restricted to cross-sectional design that precludes causal relationships. More well-designed prospective cohort studies with sexual specification are still needed.

## Data Availability Statement

The original contributions presented in the study are included in the article/Supplementary Material, further inquiries can be directed to the corresponding author/s.

## Author Contributions

YZ and JD conceived the idea, drafted this manuscript, selected and retrieved relevant papers, and assessed each study. JD performed the statistical analysis. YZ was the guarantor of the overall content. All authors revised and approved the final manuscript.

## Funding

This study was supported by National Natural Science Foundation of China (82102581), National Postdoctoral Science Foundation of China (2021M693562), Provincial Outstanding Postdoctoral Innovative Talents Program of Hunan (2021RC2020), Young Investigator Grant of Xiangya Hospital, Central South University (2020Q14), and FuQing Postdoc Program of Xiangya Hospital, Central South University (176).

## Conflict of Interest

The authors declare that the research was conducted in the absence of any commercial or financial relationships that could be construed as a potential conflictof interest.

## Publisher's Note

All claims expressed in this article are solely those of the authors and do not necessarily represent those of their affiliated organizations, or those of the publisher, the editors and the reviewers. Any product that may be evaluated in this article, or claim that may be made by its manufacturer, is not guaranteed or endorsed by the publisher.

## Supplementary Material

Supplementary Figure 1 Funnel plot with pseudo 95% confidence limits for the analysis of dietary copper intake and depression.

Supplementary Figure 2 Funnel plot with pseudo 95% confidence limits for the analysis of dietary selenium intake and depression.

Supplementary Figure 3 Funnel plot with pseudo 95% confidence limits for the analysis of dietary manganese intake and depression.
