# Supplementary figures and images for: Associations of Dietary Copper, Selenium, and Manganese Intake With Depression: A Meta-Analysis of Observational Studies
Source: Front Nutr. 2022 Mar 15;9:854774. doi: 10.3389/fnut.2022.854774 (PMC8965358; doi:10.3389/fnut.2022.854774)

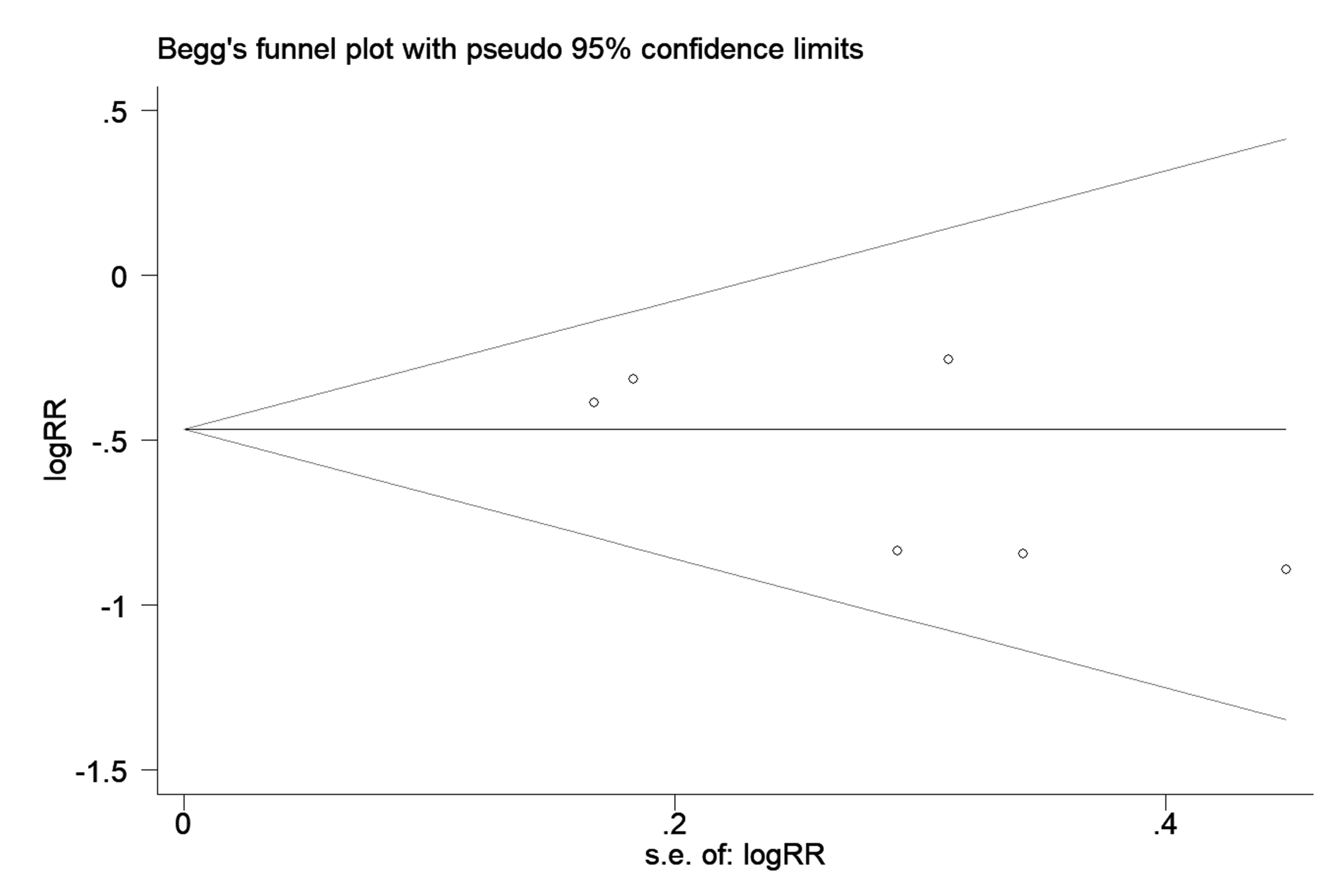

Supplement: Supplementary Figure 1 — Funnel plot with pseudo 95% confidence limits for the analysis of dietary copper intake and depression. [file Image_1.TIF]

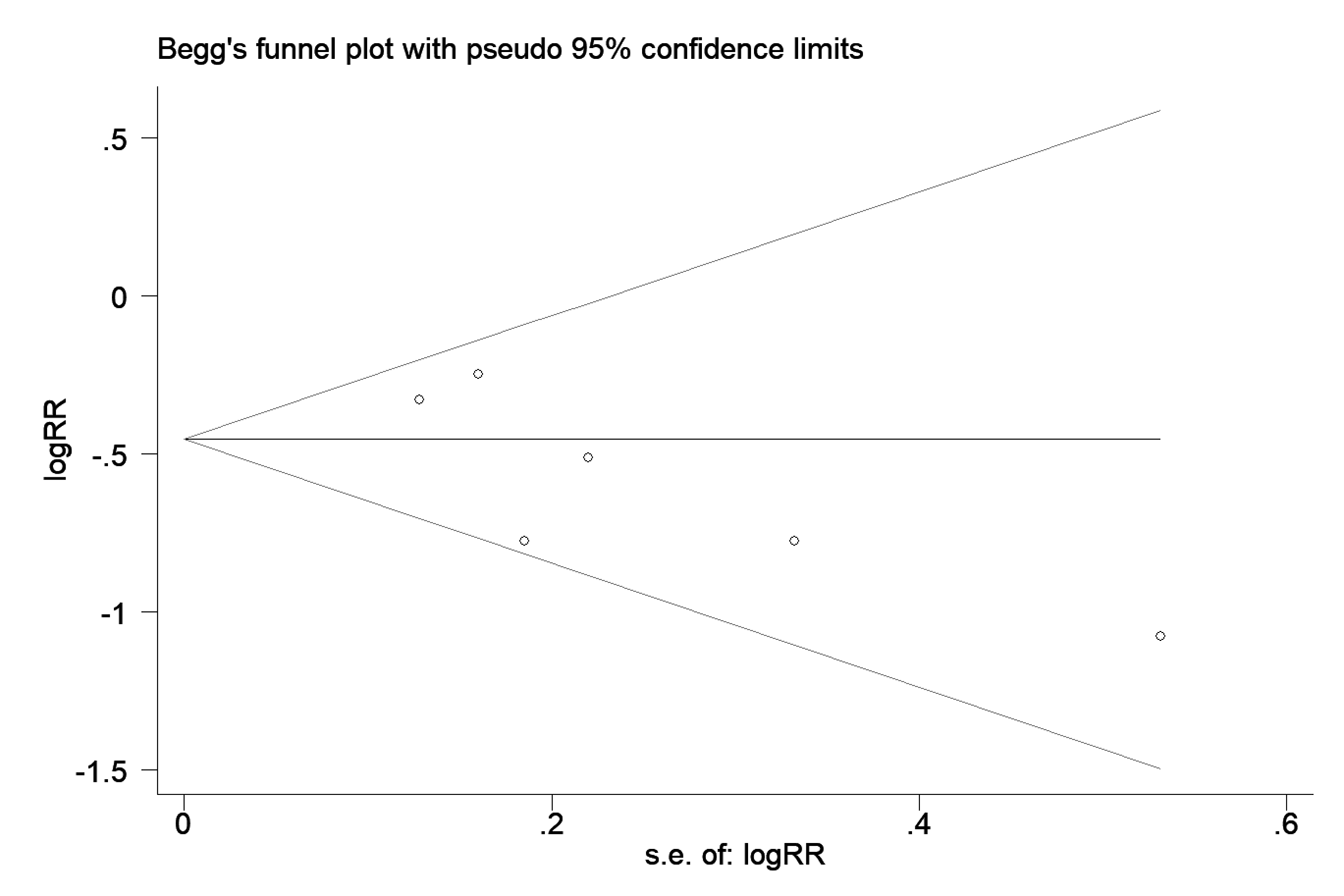

Supplement: Supplementary Figure 2 — Funnel plot with pseudo 95% confidence limits for the analysis of dietary selenium intake and depression. [file Image_2.TIF]

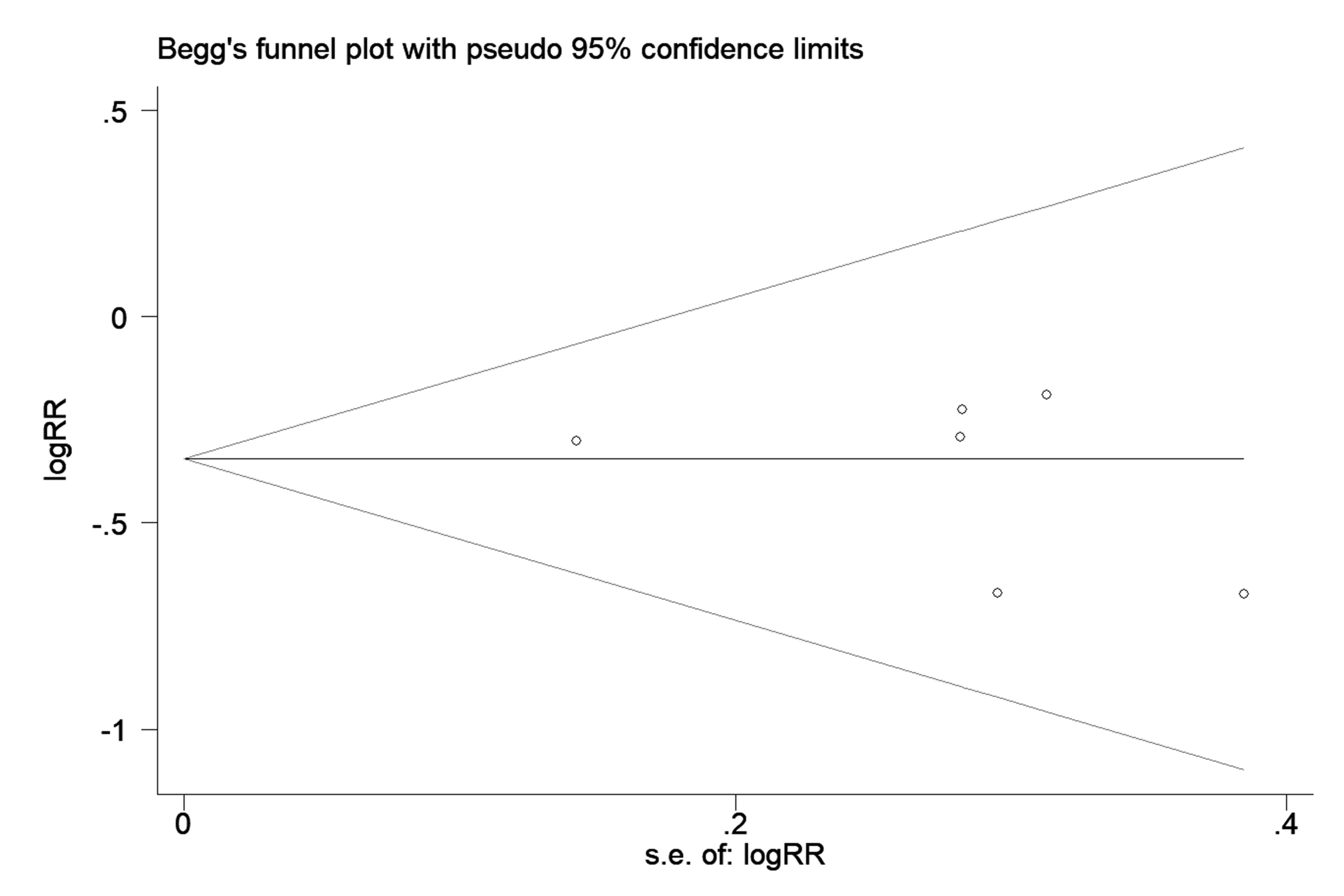

Supplement: Supplementary Figure 3 — Funnel plot with pseudo 95% confidence limits for the analysis of dietary manganese intake and depression. [file Image_3.TIF]
